# Supplementary material for: Microbiome gut community structure and functionality are associated with symptom severity in non-responsive celiac disease patients undergoing a gluten-free diet
Source: mSystems. 2025 Jun 6;10(7):e00143-25. doi: 10.1128/msystems.00143-25 (PMC12282095; doi:10.1128/msystems.00143-25)
Supplement: Supplemental Figures — Figures S1 to S3 [file msystems.00143-25-s0005.docx]

**Supplementary Figure 1:** PRISMA flowchart summarizing the process to select metagenomics publicly available data from CD asymptomatic patients .

**
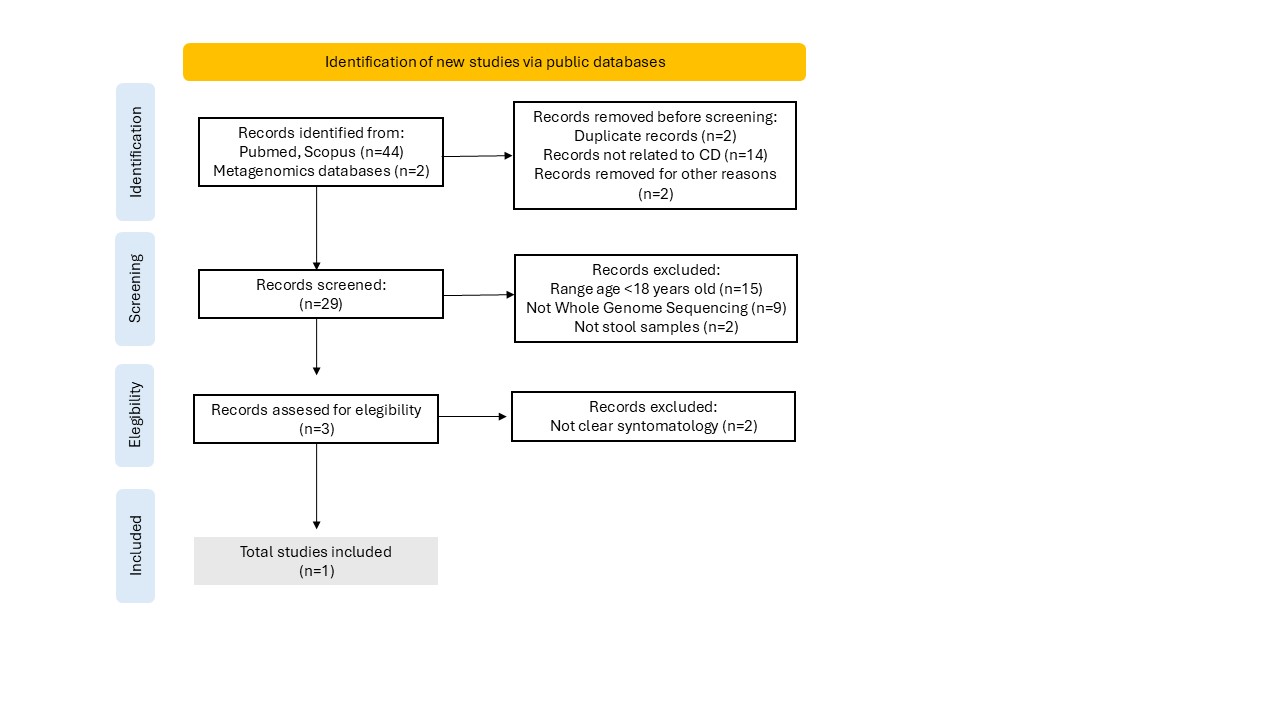
**

Initial search was made using PubMed and SCOPUS databases with the following search terms: (((celiac disease) AND (metagenomics)) NOT (mice)) NOT (review[Publication Type]) and metagenomics repositories (MGNify [24] and curatedMetagenomicData [25]). In the identification phase: Records not related to CD included Crohn’s Disease. Records removed for other reasons included one review, and one study conducted on primates.

**Supplementary Figure 2A:** Plots of variables in multiple factor analysis (A) Group representation and (B) Circle plot showing the quantitative variables contribution.


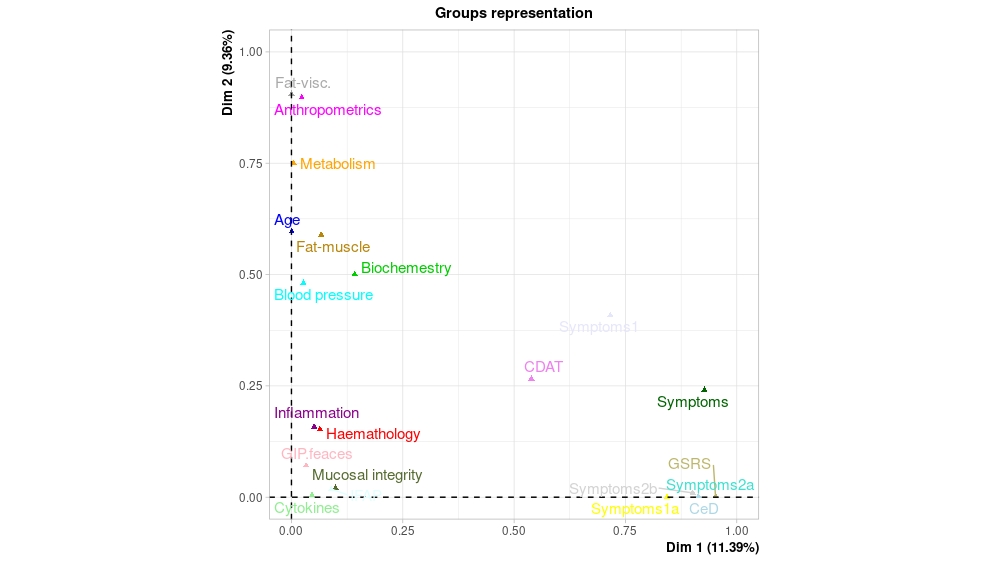

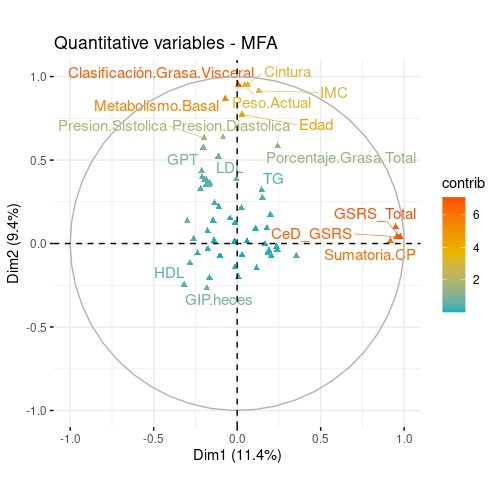


**A**

**B**

Variables clustered closely indicate positive correlations, while those projecting in opposite directions suggest negative correlations. The group factors map (A) illustrates categorical variable interrelationships, and the correlation circle plot (B) shows continuous variable interrelationships. Cos2 values quantify each variable's contribution to the factor map, with variables nearer to the plot center having lower weightage.

**Supplementary Figure 2B.** Scree plot to select the variables to retain (A), and contribution of variables to dimension one (B), two (C) and three (D).


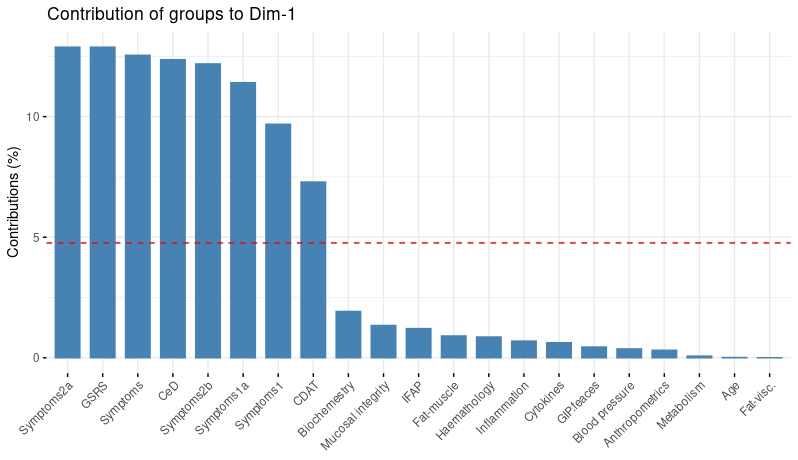

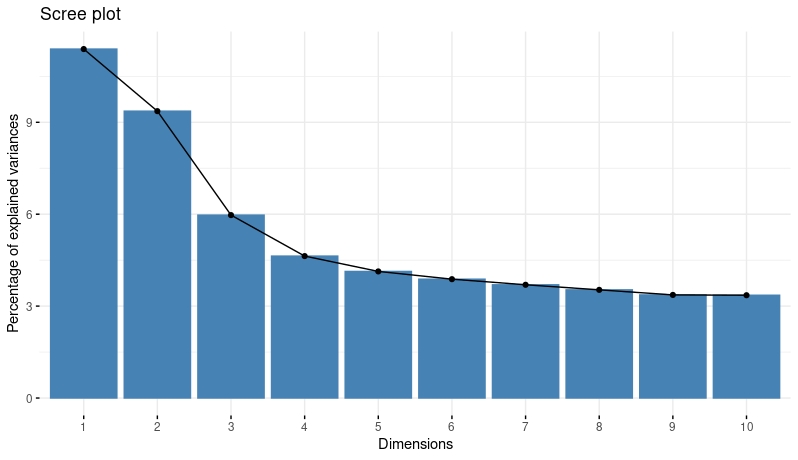


**B**

**A**


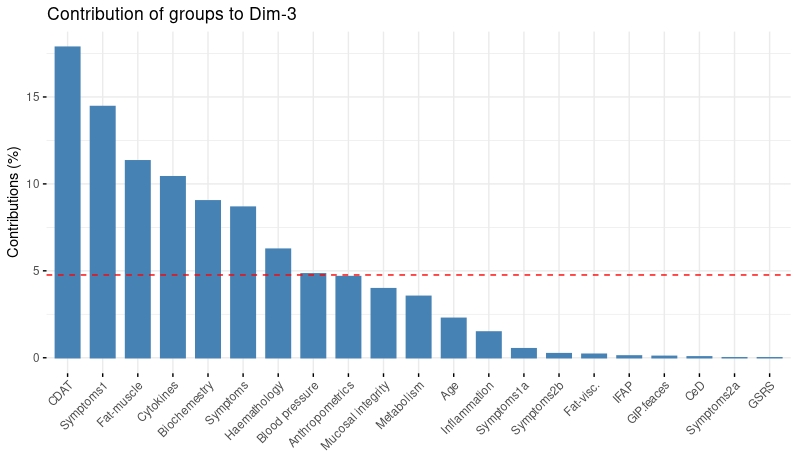

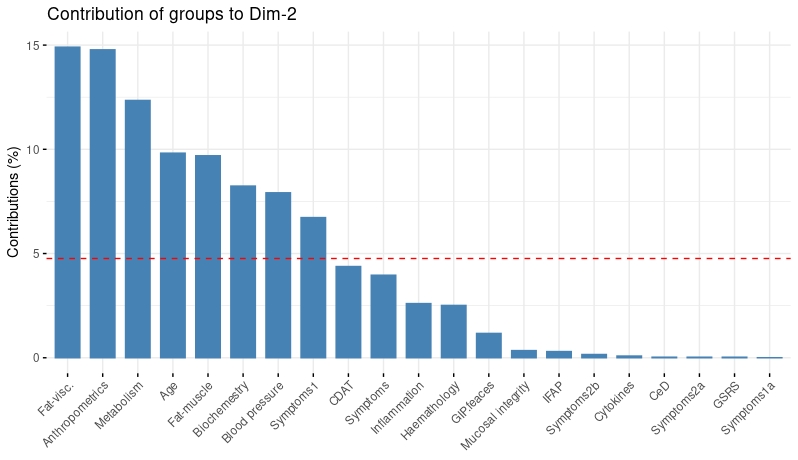


**D**

**C**

According to the scree test, the elbow of the graph where the eigenvalues seem to level off is 3 indicating that components to the left of this point should be retained as significant. In each dimension variables showing a contribution greater than 5% were retained for analysis.

**Supplementary Figure 3** (A) Multidimensional scaling (MDS) of the Unifrac distance representing beta diversity before batch effect correction of the samples (B) Same as A but after batch effect correction.


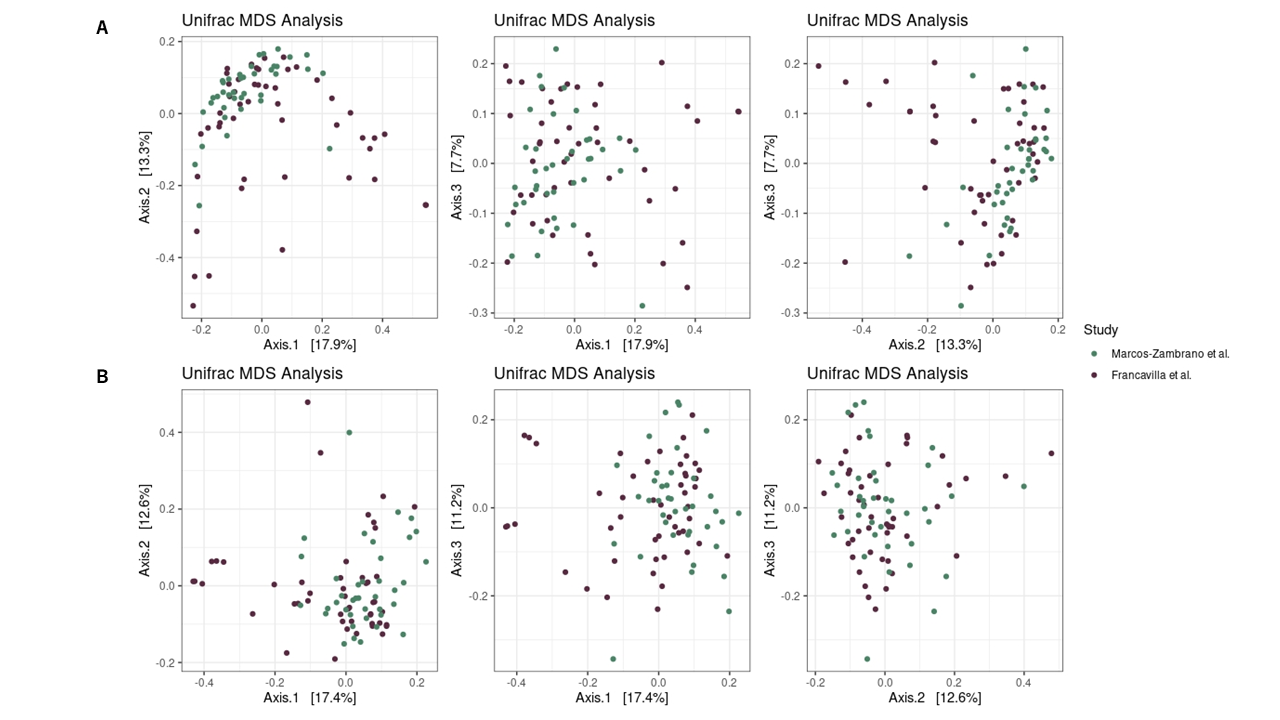


Francavilla et al. corresponding to the public dataset used, and Marcos-Zambrano et al. corresponding to the NRCD patient’s cohort from this study.
