# Supplementary material for: Microbiome gut community structure and functionality are associated with symptom severity in non-responsive celiac disease patients undergoing a gluten-free diet
Source: mSystems. 2025 Jun 6;10(7):e00143-25. doi: 10.1128/msystems.00143-25 (PMC12282095; doi:10.1128/msystems.00143-25)

Supplementary figure 4A: Sankey Network diagram showing the identified microbes in the samples of the gut microbiome of patients from the study and their connection with the significant microbial metabolic pathways after MPEA analysis and the reactions involved.

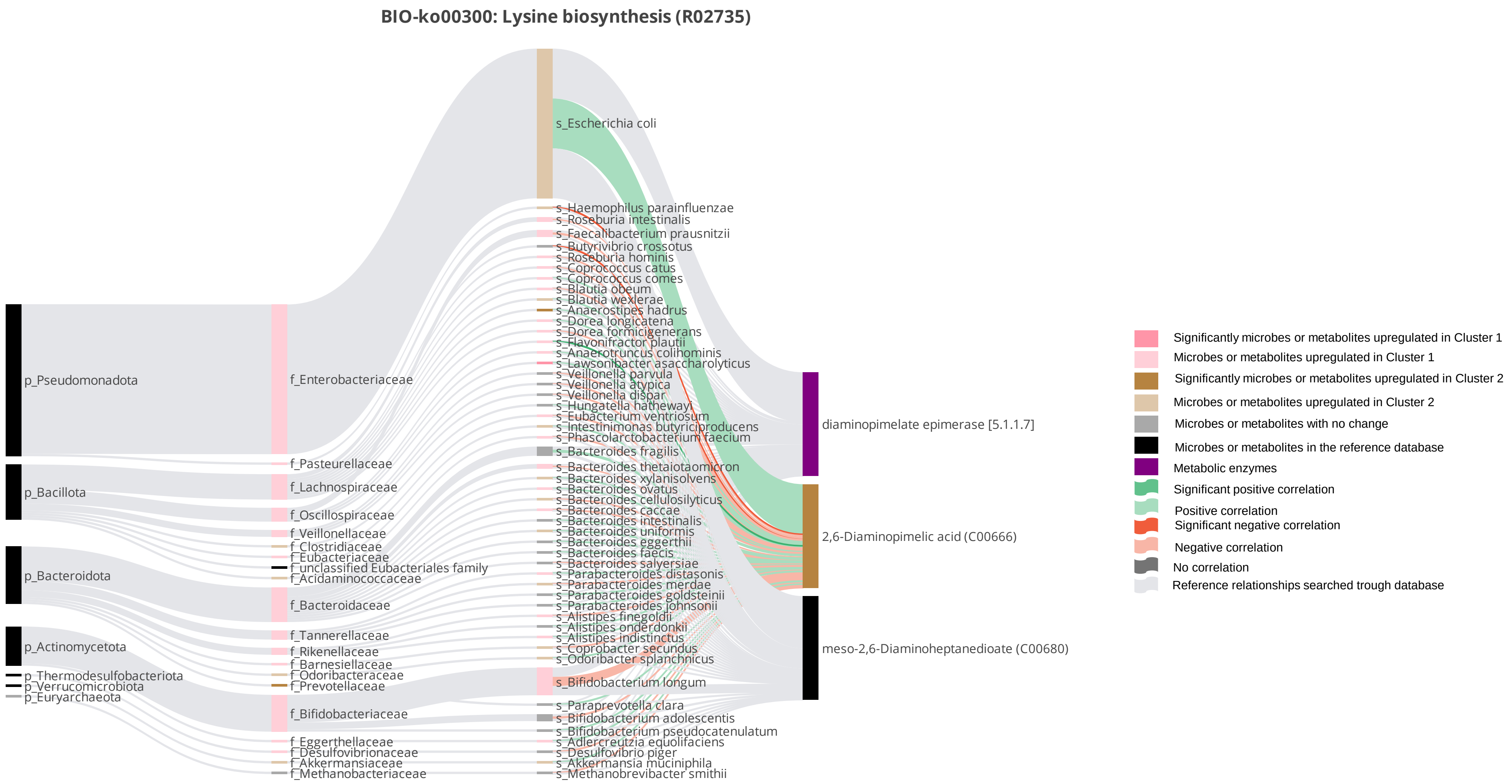

Supplementary figure 4B: Sankey Network diagram showing the identified microbes in the samples of the gut microbiome of patients from the study and their connection with the significant metabolic pathways from microbial and hos co-metabolism after MPEA analysis and the reactions involved.

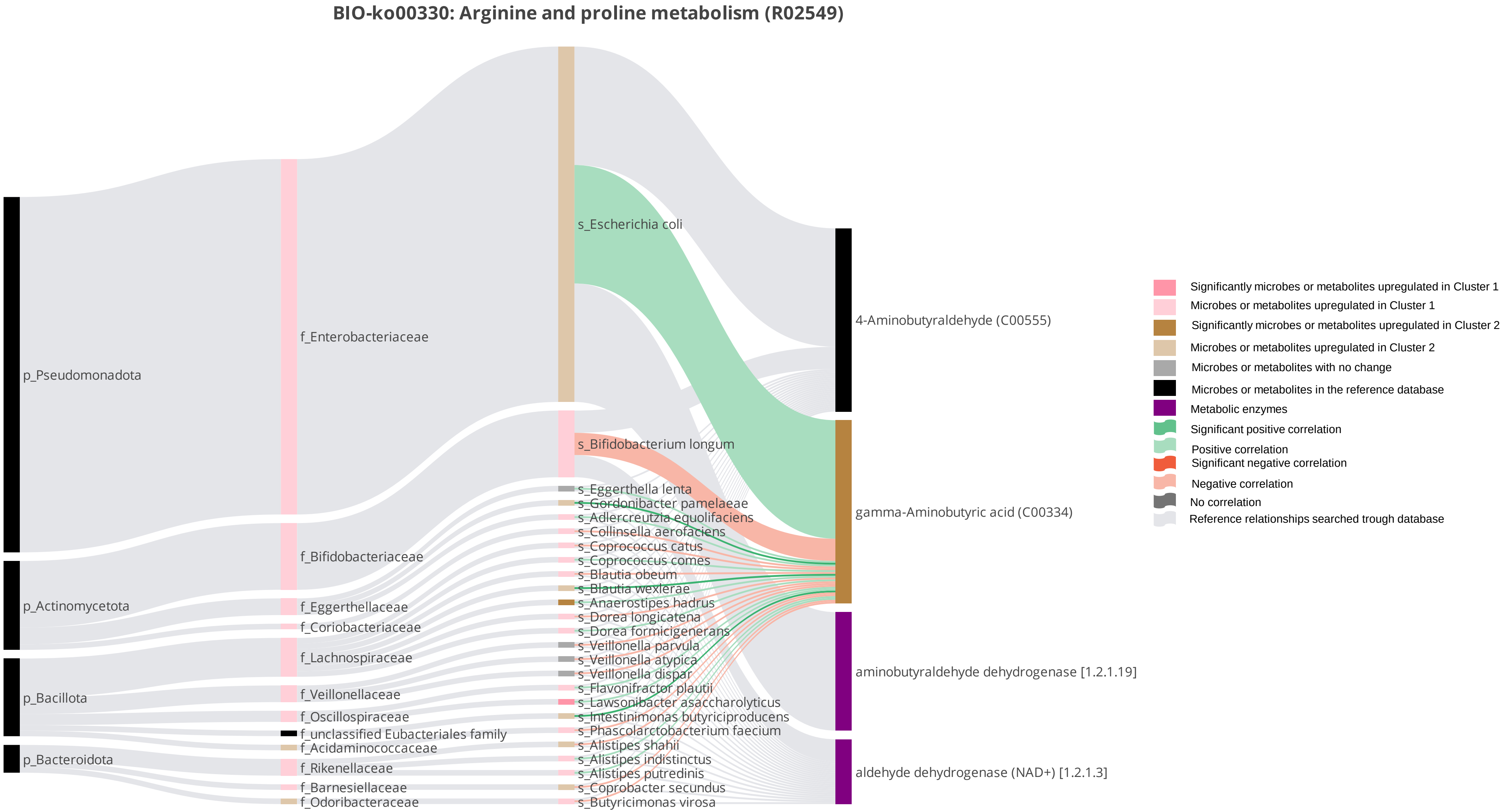

Supplementary figure 4C: Sankey Network diagram showing the identified microbes in the samples of the gut microbiome of patients from the study and their connection with the significant metabolic pathways from microbial and hos co-metabolism after MPEA analysis and the reactions involved.

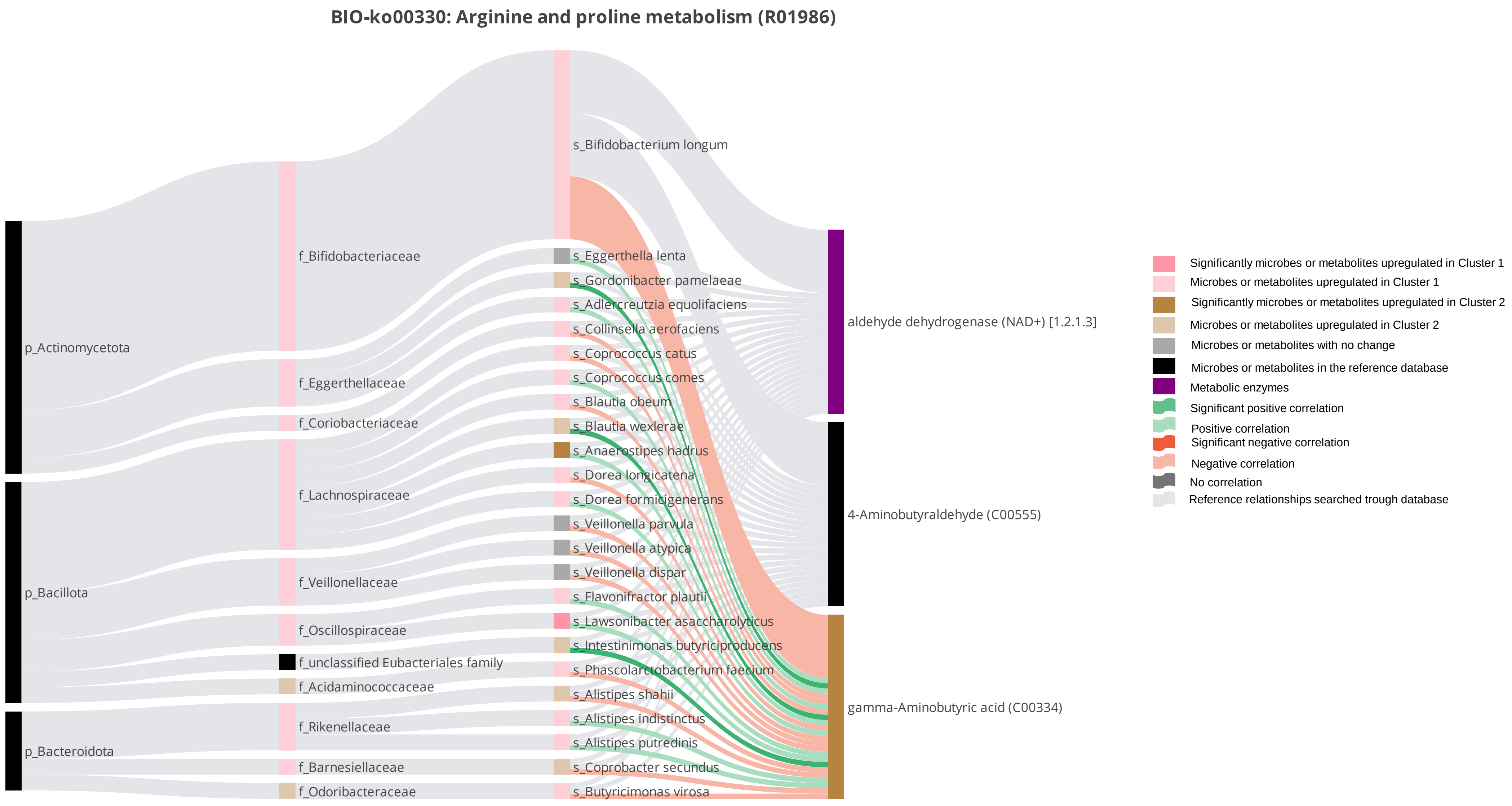

Supplementary figure 4D: Sankey Network diagram showing the identified microbes in the samples of the gut microbiome of patients from the study and their connection with the significant metabolic pathways from microbial and hos co-metabolism after MPEA analysis and the reactions involved.

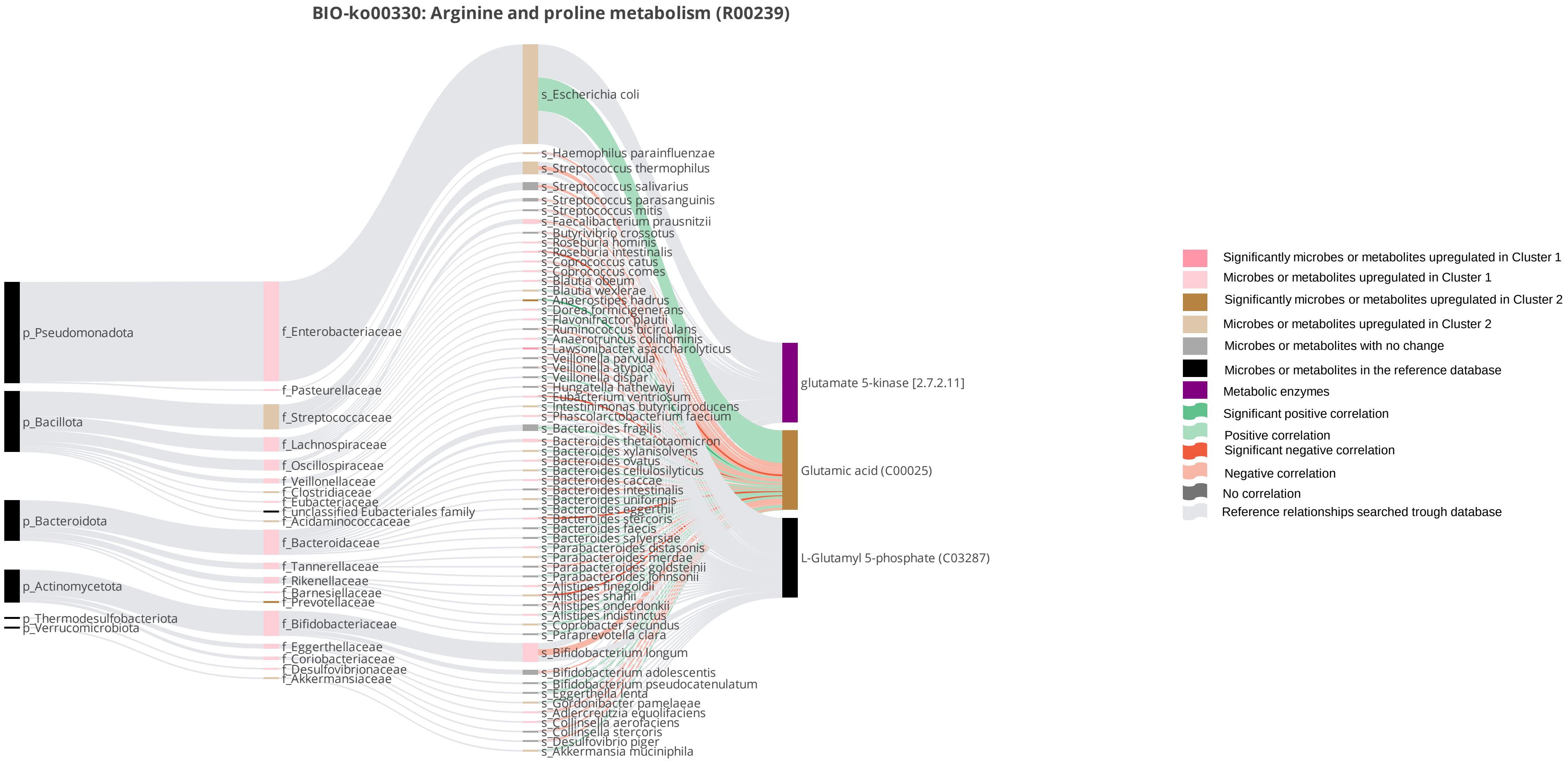

Supplementary figure 4E: Sankey Network diagram showing the identified microbes in the samples of the gut microbiome of patients from the study and their connection with the significant metabolic pathways from microbial and hos co-metabolism after MPEA analysis and the reactions involved.

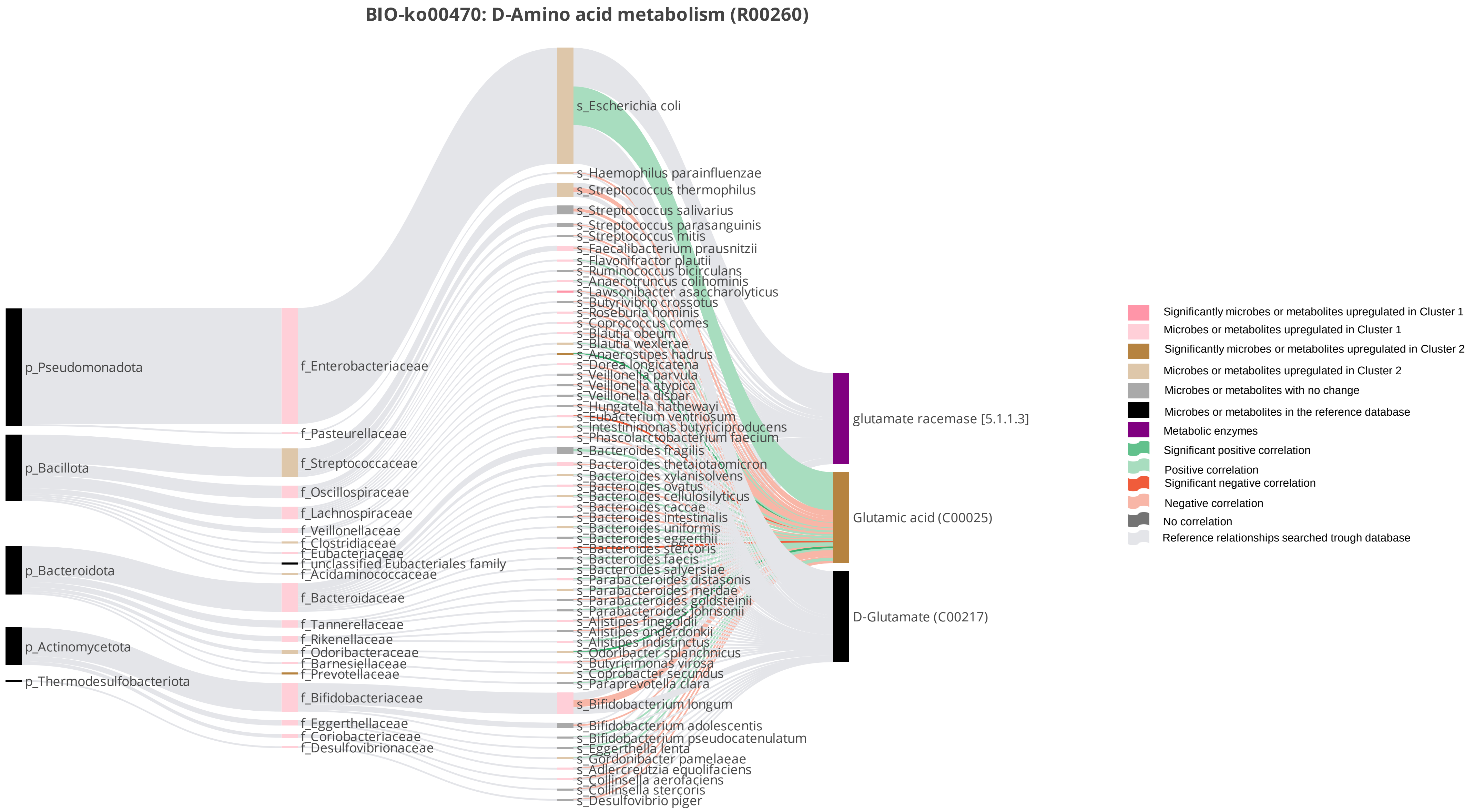

Supplementary figure 4F: Sankey Network diagram showing the identified microbes in the samples of the gut microbiome of patients from the study and their connection with the significant metabolic pathways from microbial and hos co-metabolism after MPEA analysis and the reactions involved.

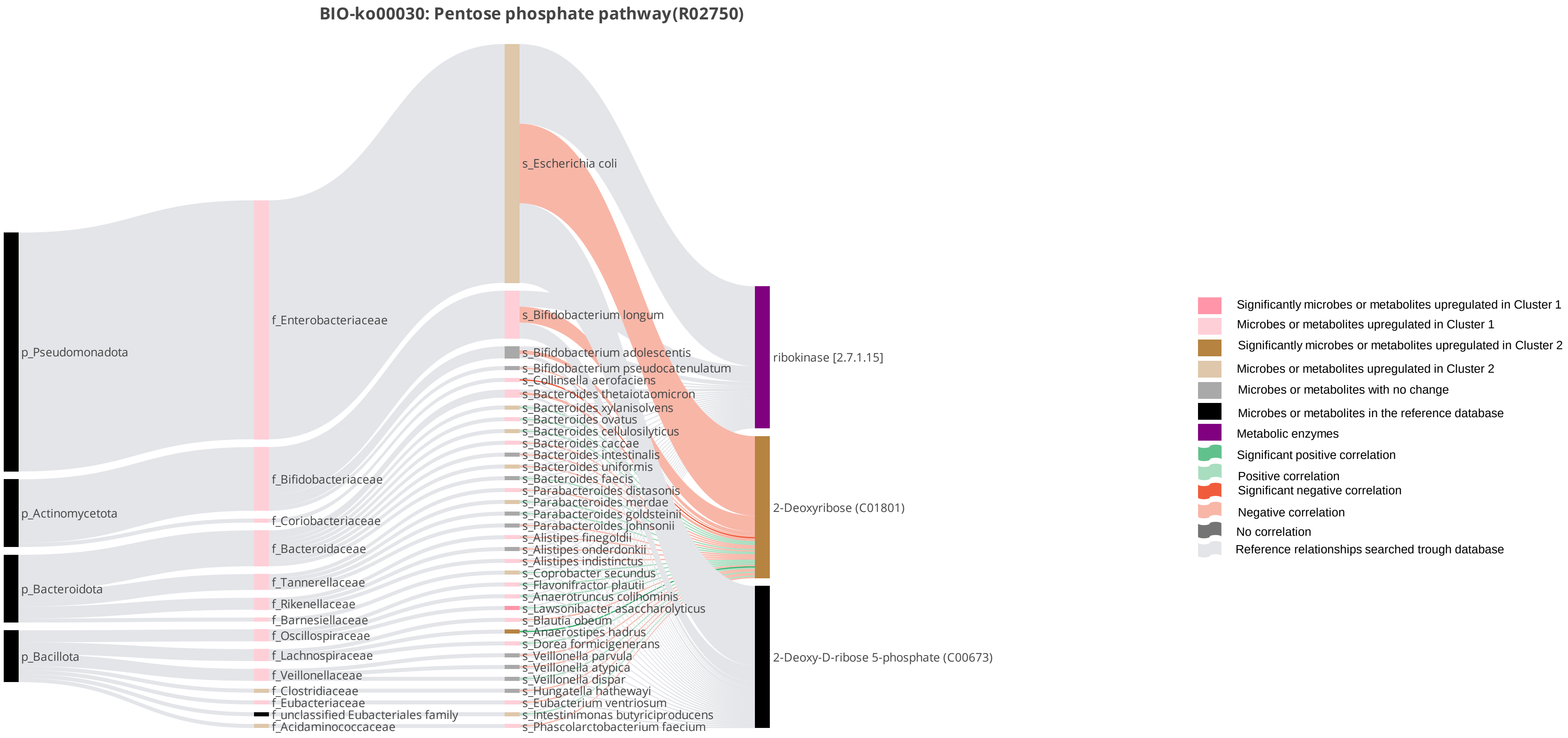

Supplementary figure 4G: Sankey Network diagram showing the identified microbes in the samples of the gut microbiome of patients from the study and their connection with the significant metabolic pathways from microbial and hos co-metabolism after MPEA analysis and the reactions involved.

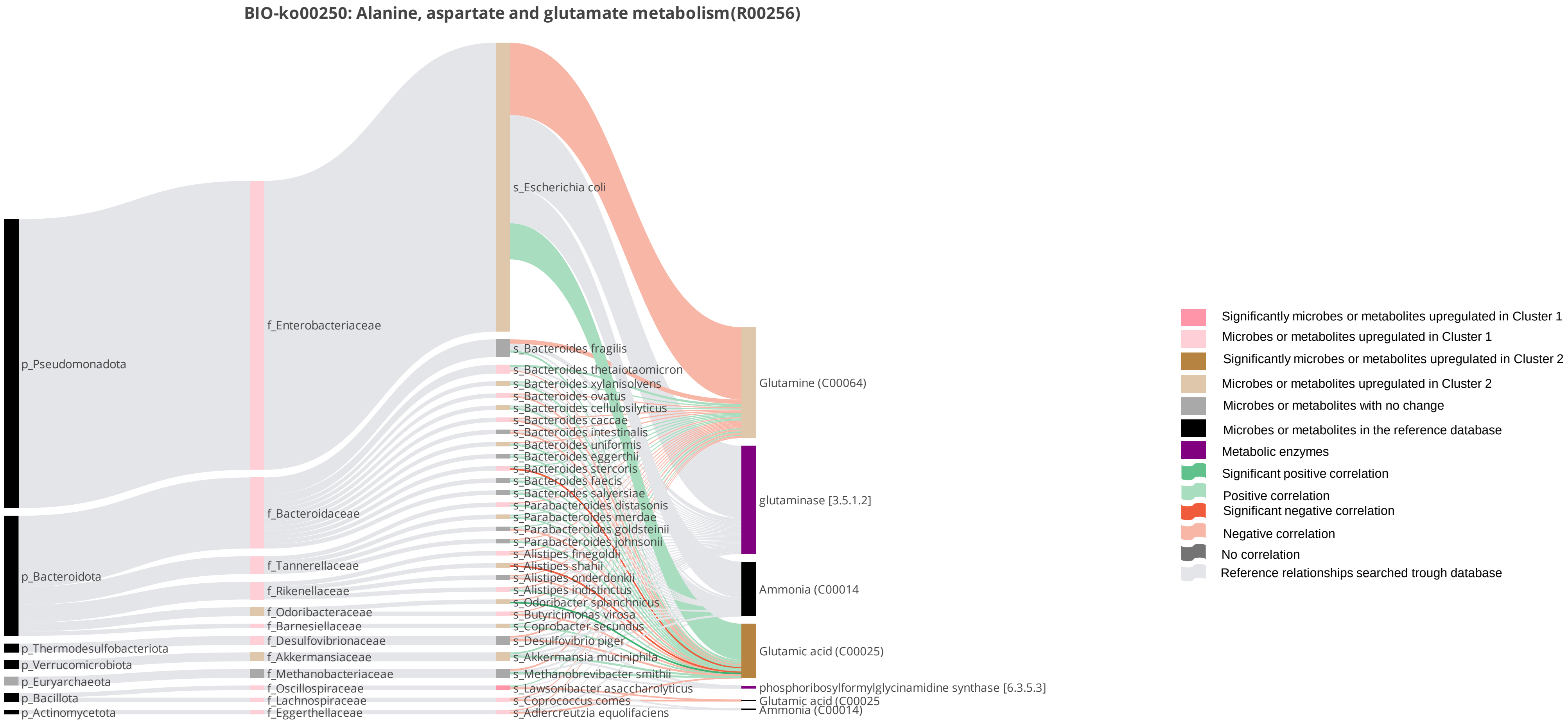

Supplementary figure 4H: Sankey Network diagram showing the identified microbes in the samples of the gut microbiome of patients from the study and their connection with the significant metabolic pathways from microbial and hos co-metabolism after MPEA analysis and the reactions involved.

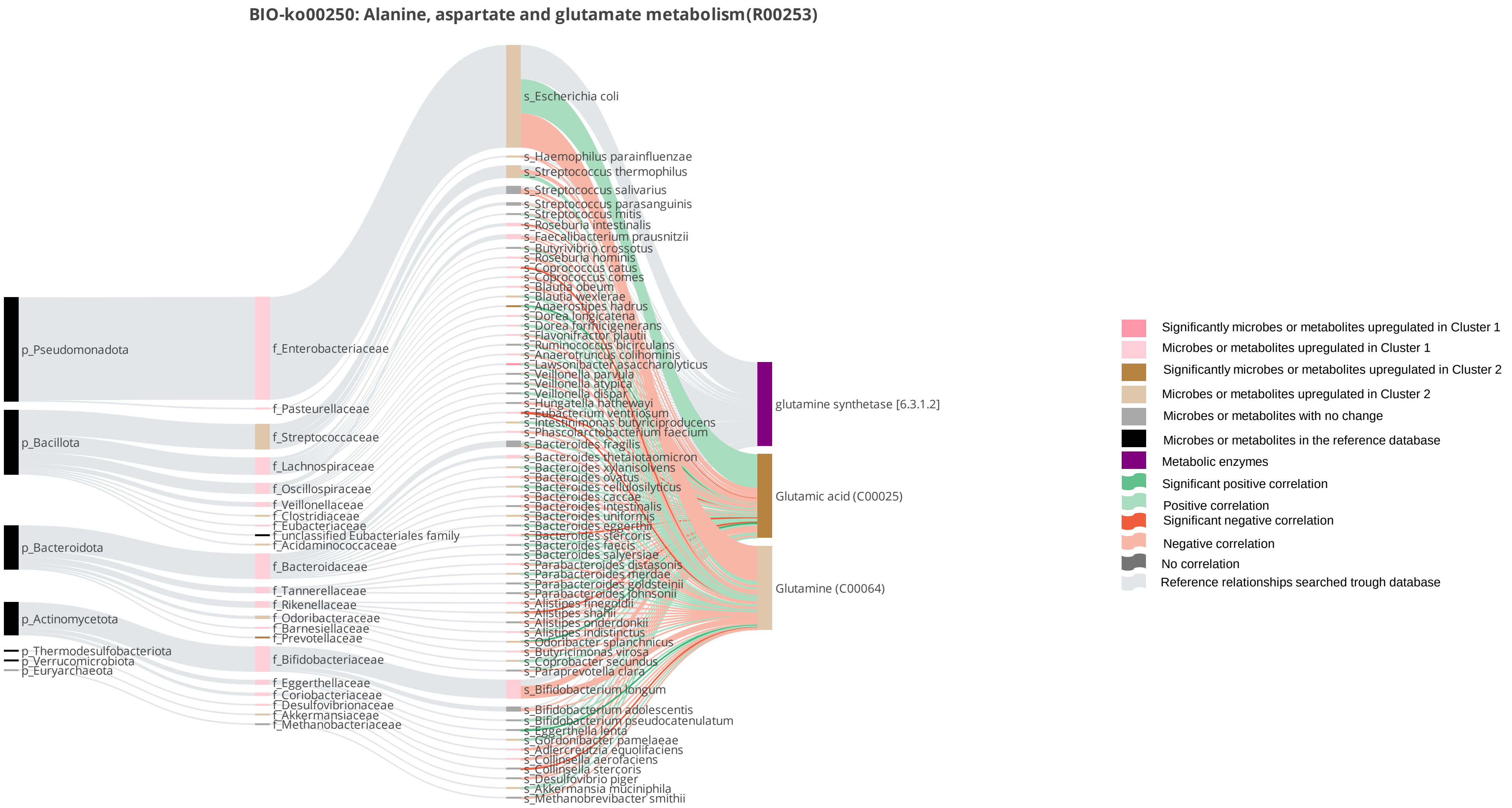

Supplementary figure 4I: Sankey Network diagram showing the identified microbes in the samples of the gut microbiome of patients from the study and their connection with the significant metabolic pathways from microbial and hos co-metabolism after MPEA analysis and the reactions involved.

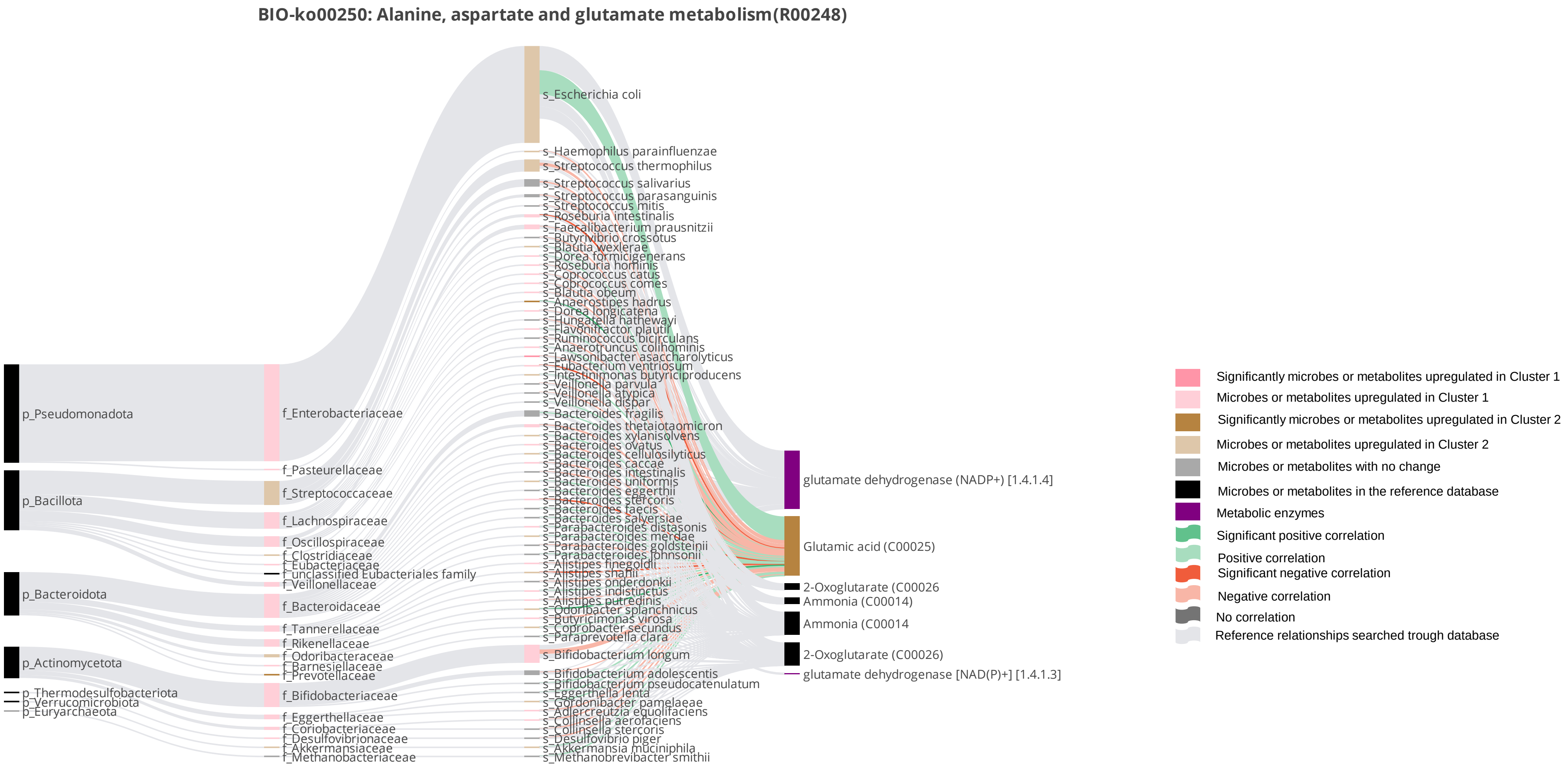

Supplementary figure 4J: Sankey Network diagram showing the identified microbes in the samples of the gut microbiome of patients from the study and their connection with the significant metabolic pathways from microbial and hos co-metabolism after MPEA analysis and the reactions involved.

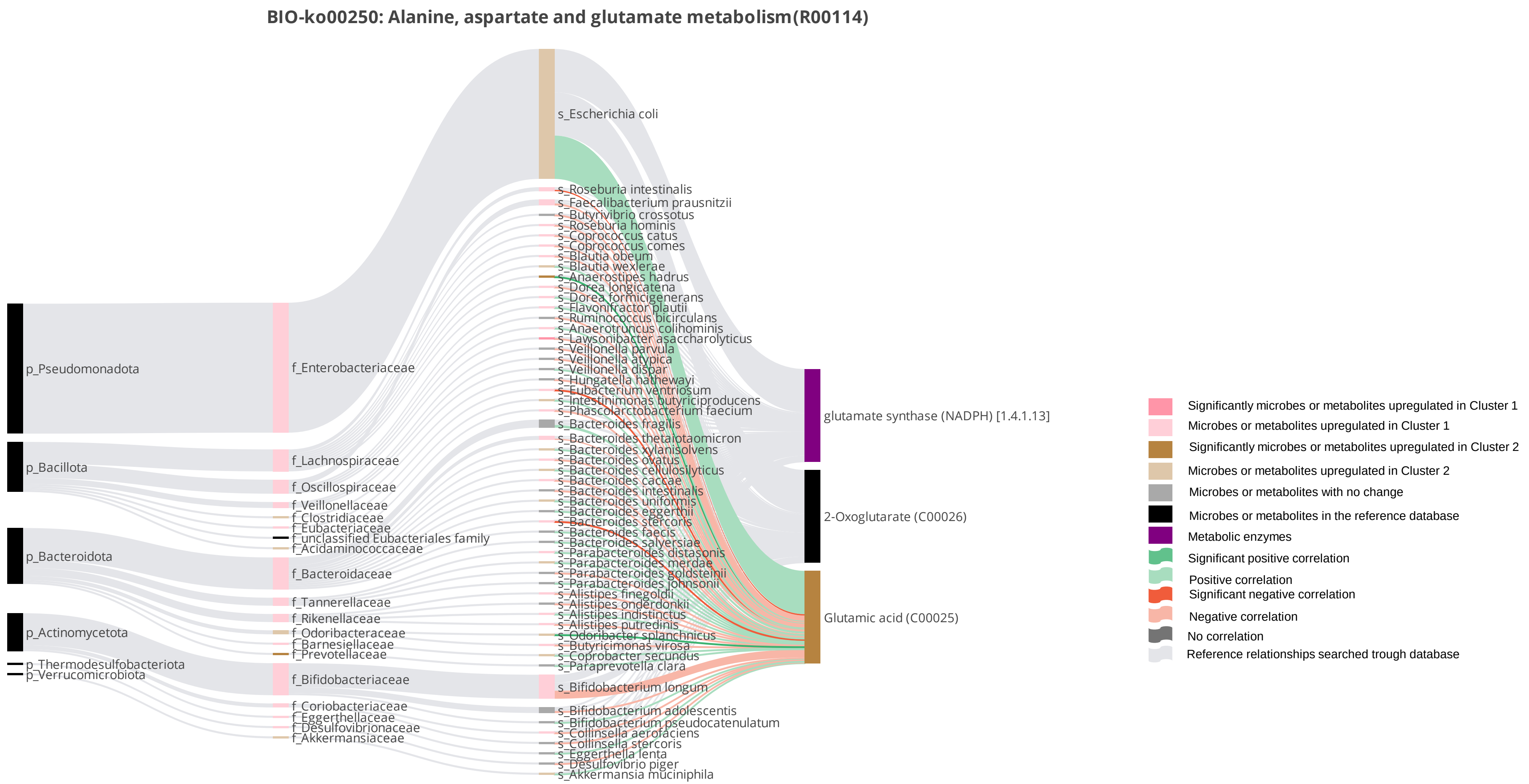

Supplementary figure 4K: Sankey Network diagram showing the identified microbes in the samples of the gut microbiome of patients from the study and their connection with the significant metabolic pathways from microbial and hos co-metabolism after MPEA analysis and the reactions involved.

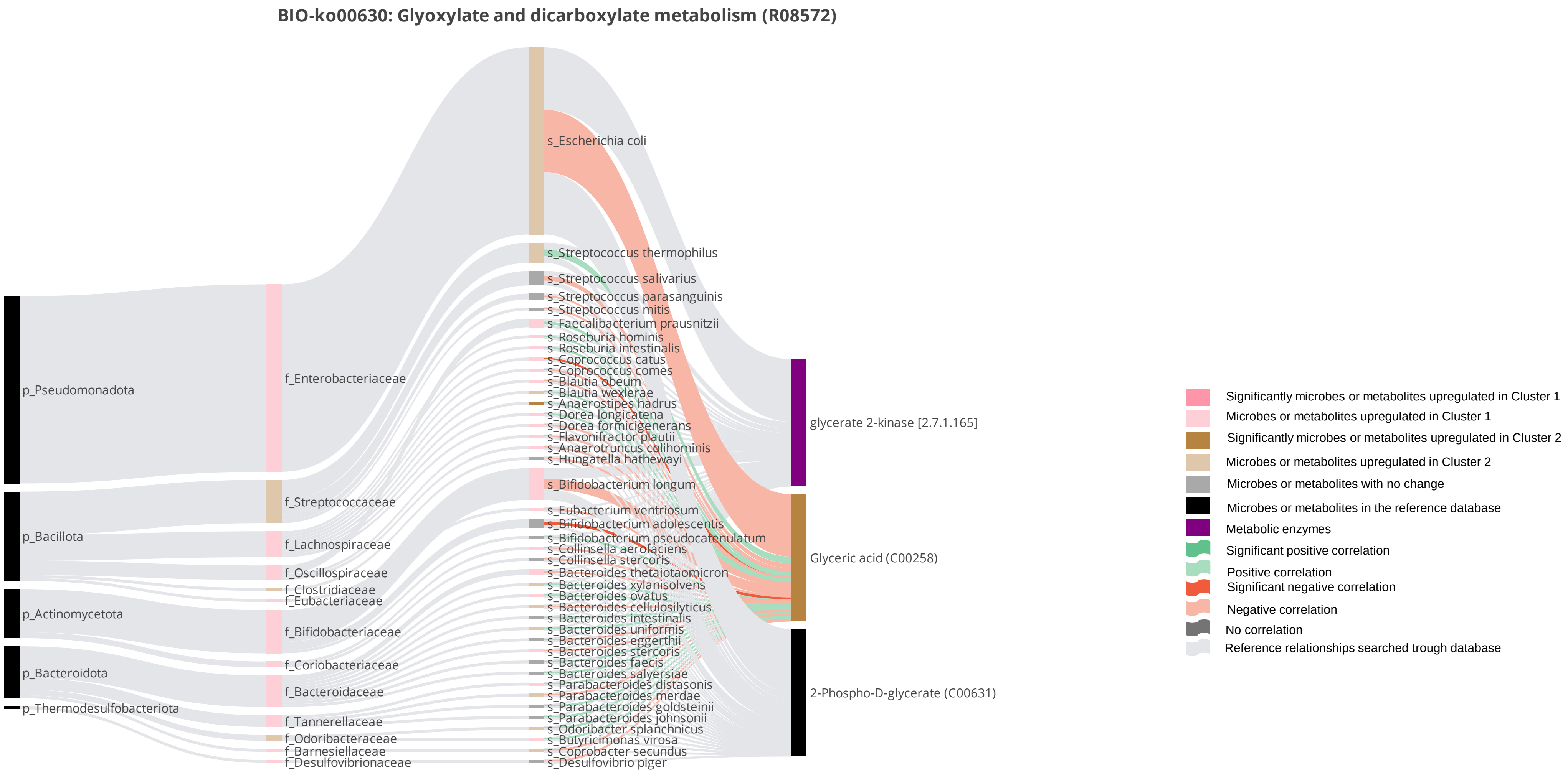

Supplementary figure 4L: Sankey Network diagram showing the identified microbes in the samples of the gut microbiome of patients from the study and their connection with the significant metabolic pathways from microbial and hos co-metabolism after MPEA analysis and the reactions involved.

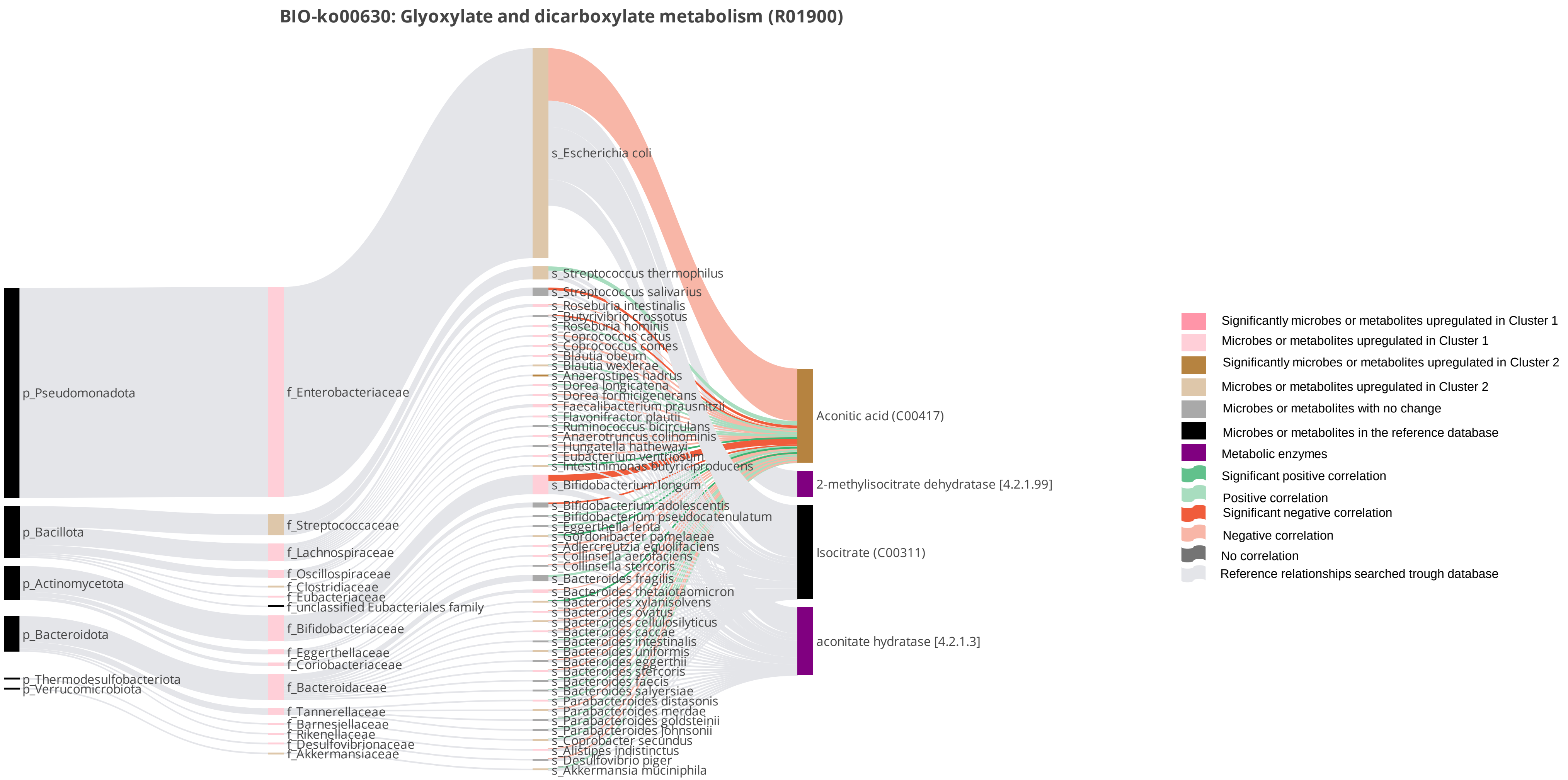

Supplementary figure 4M: Sankey Network diagram showing the identified microbes in the samples of the gut microbiome of patients from the study and their connection with the significant metabolic pathways from microbial and hos co-metabolism after MPEA analysis and the reactions involved.

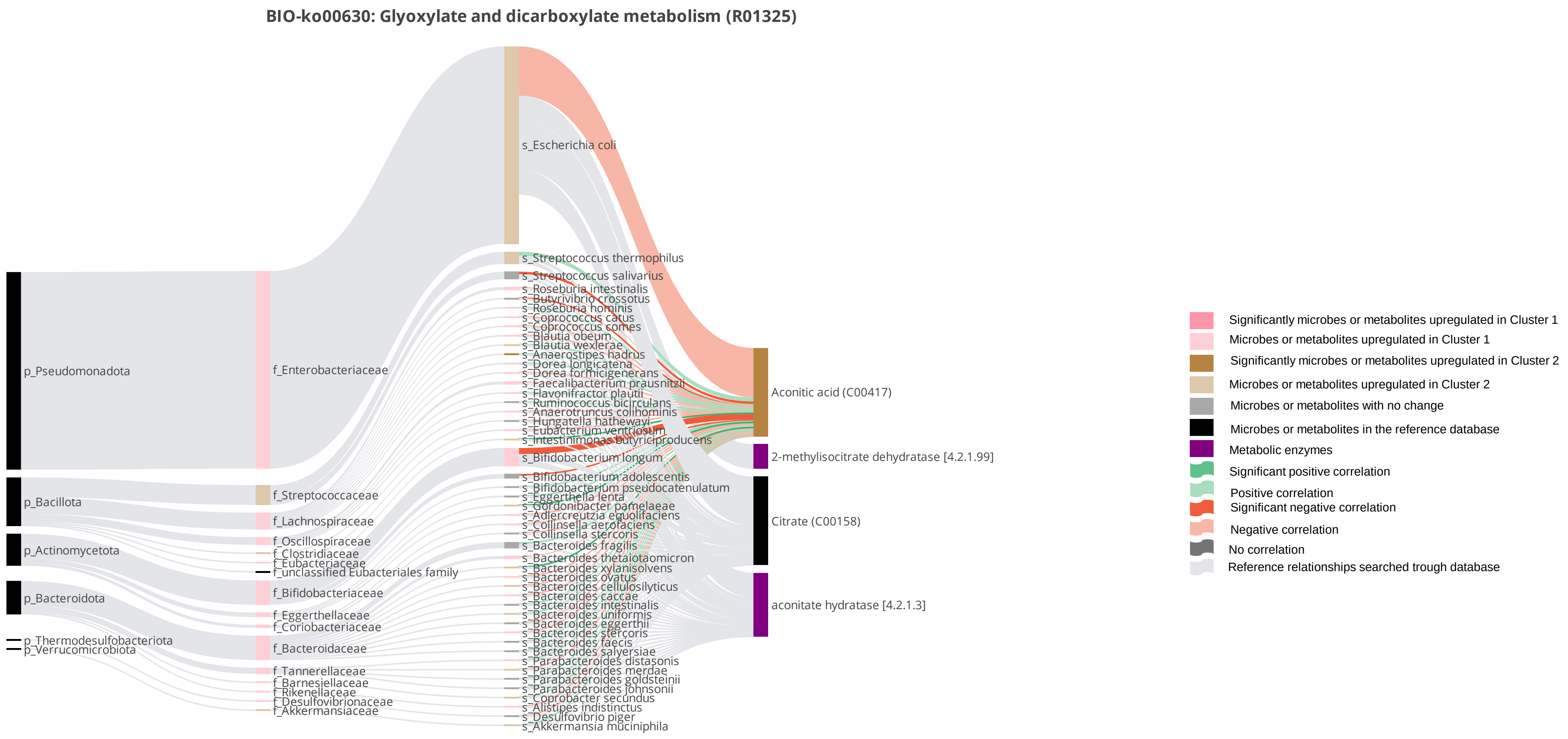

Supplementary figure 4N: Sankey Network diagram showing the identified microbes in the samples of the gut microbiome of patients from the study and their connection with the significant metabolic pathways from microbial and hos co-metabolism after MPEA analysis and the reactions involved.

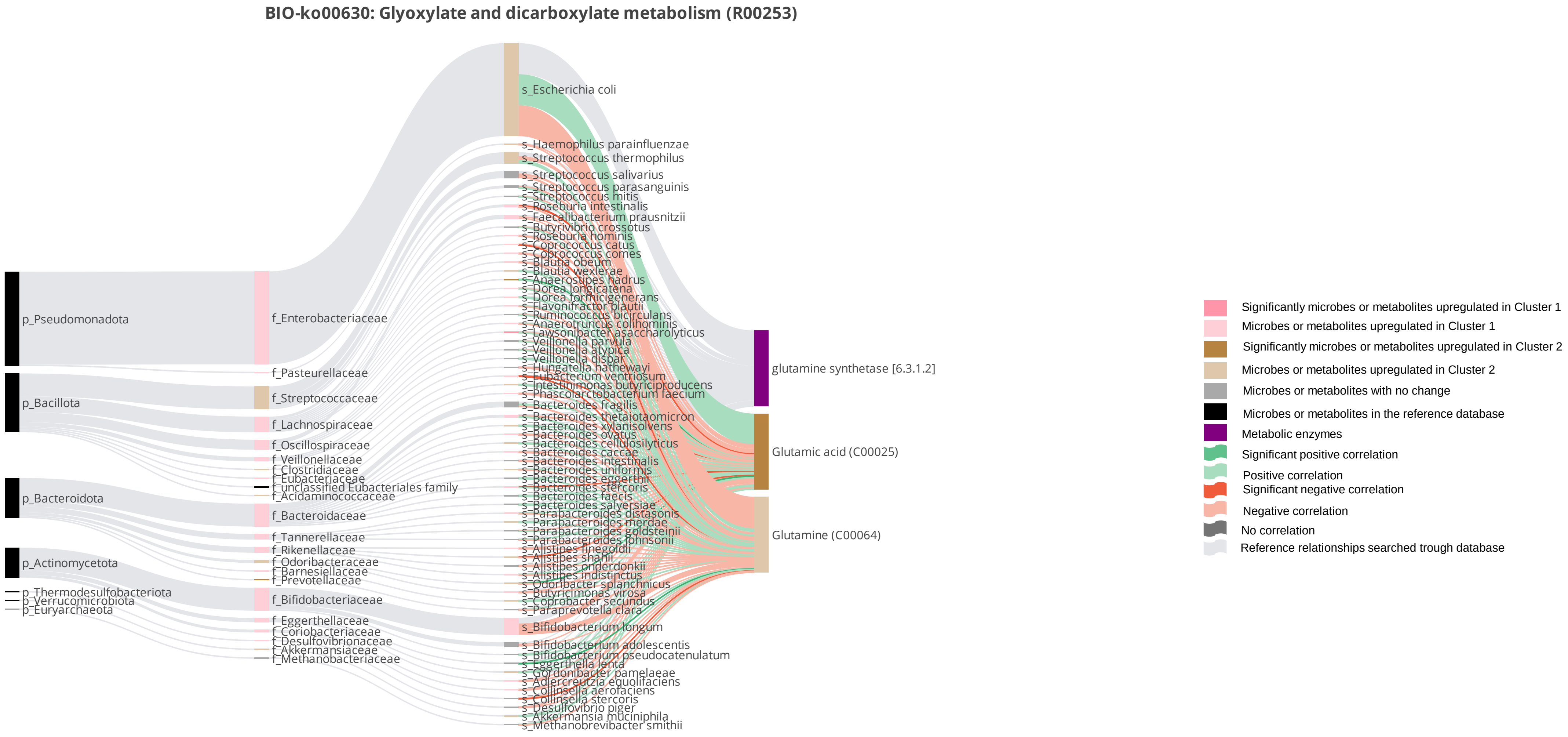

Supplementary figure 4O: Sankey Network diagram showing the identified microbes in the samples of the gut microbiome of patients from the study and their connection with the significant metabolic pathways from microbial and hos co-metabolism after MPEA analysis and the reactions involved.

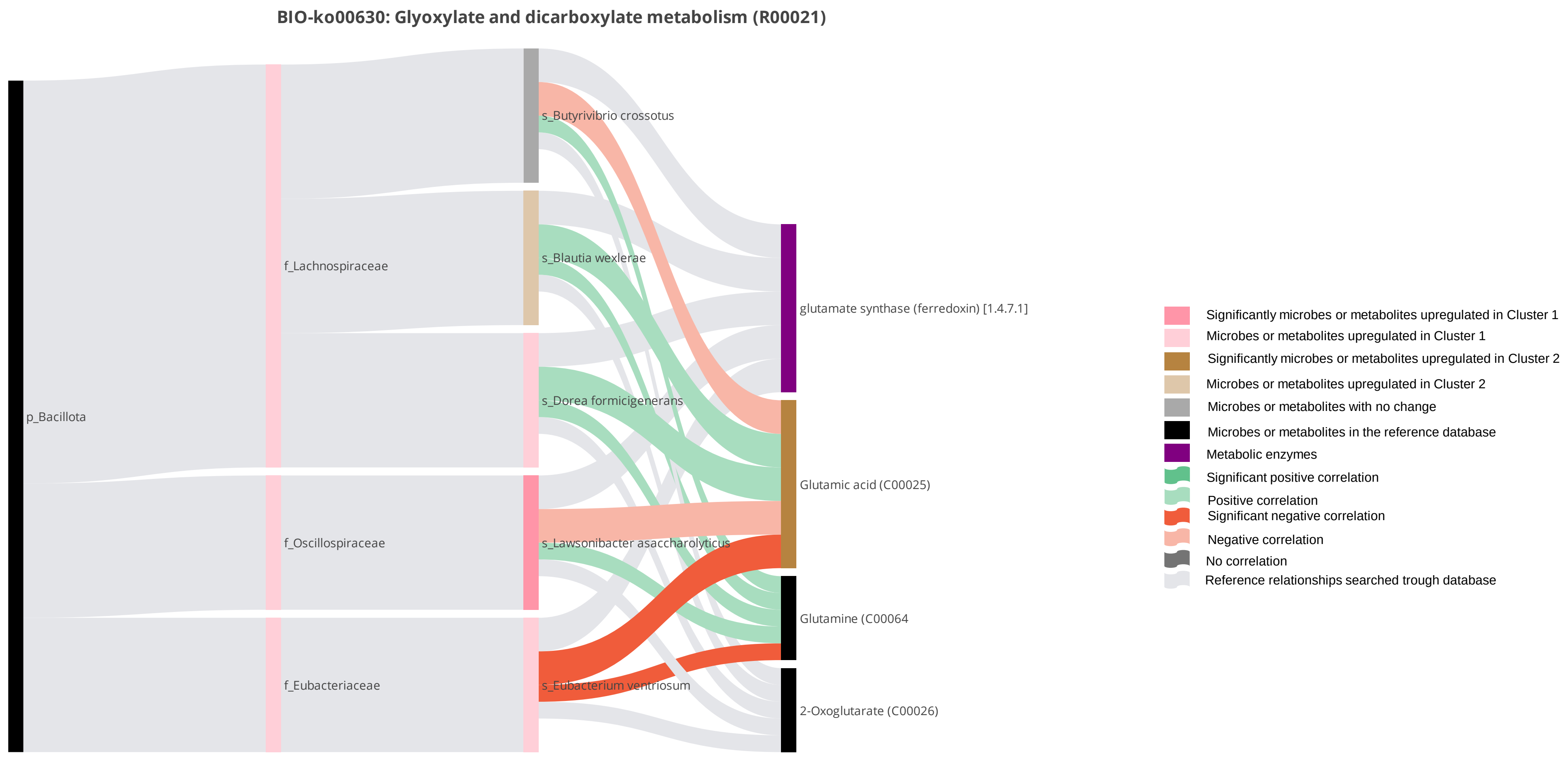

Supplementary figure 4P: Sankey Network diagram showing the identified microbes in the samples of the gut microbiome of patients from the study and their connection with the significant metabolic pathways from microbial and hos co-metabolism after MPEA analysis and the reactions involved.

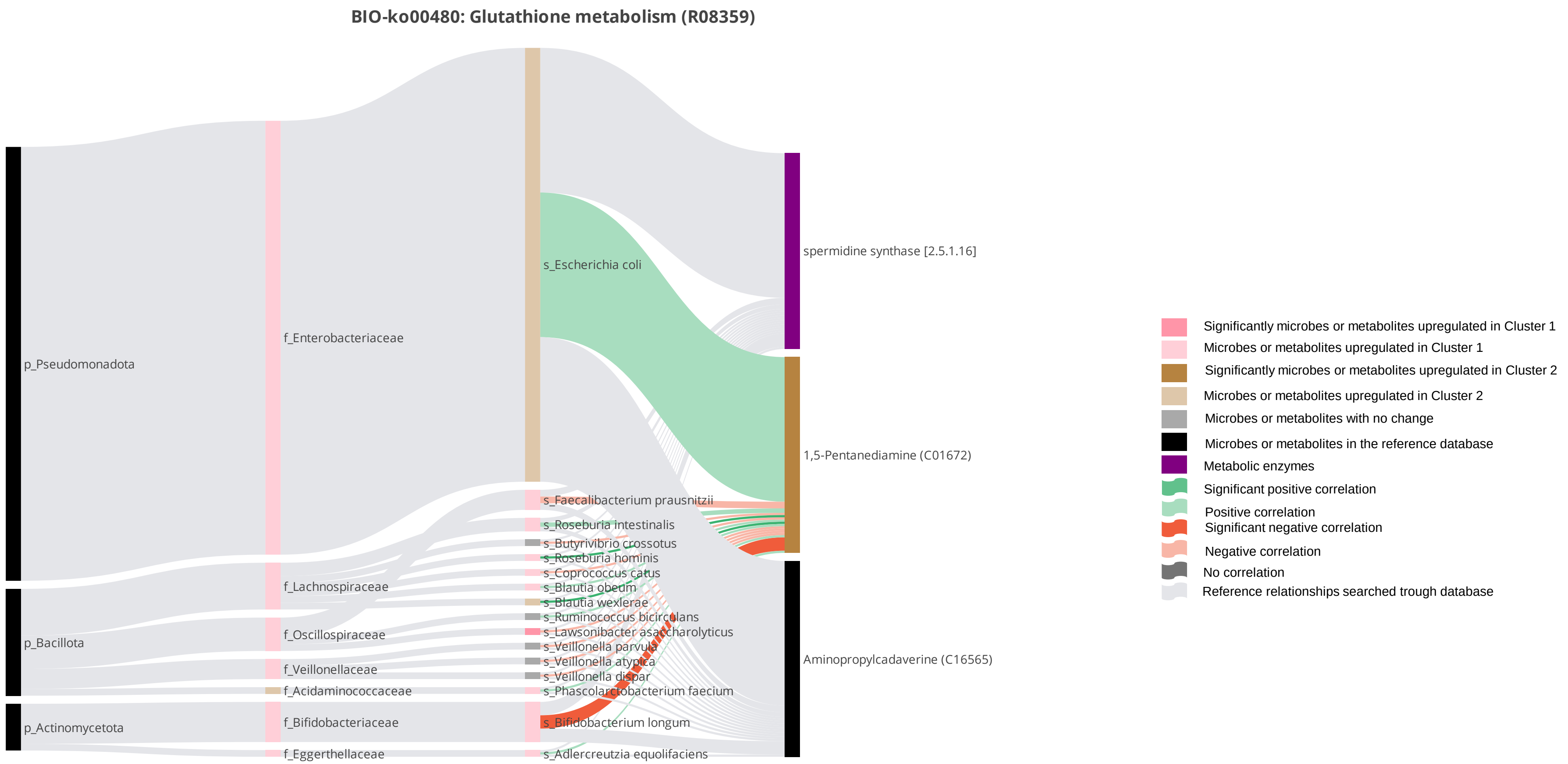

Supplementary figure 4Q: Sankey Network diagram showing the identified microbes in the samples of the gut microbiome of patients from the study and their connection with the significant metabolic pathways from microbial and hos co-metabolism after MPEA analysis and the reactions involved.

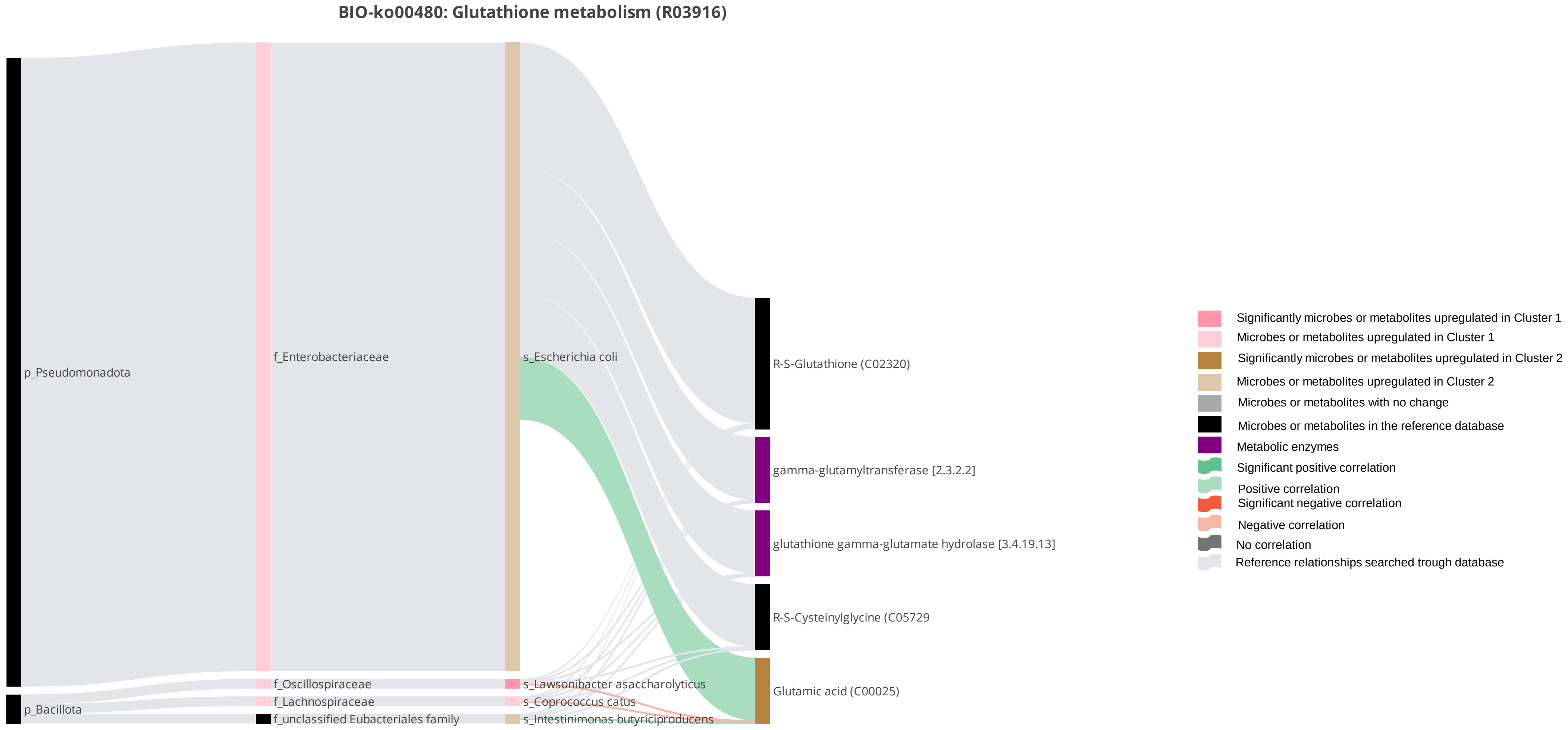

Supplementary figure 4R: Sankey Network diagram showing the identified microbes in the samples of the gut microbiome of patients from the study and their connection with the significant metabolic pathways from microbial and hos co-metabolism after MPEA analysis and the reactions involved.

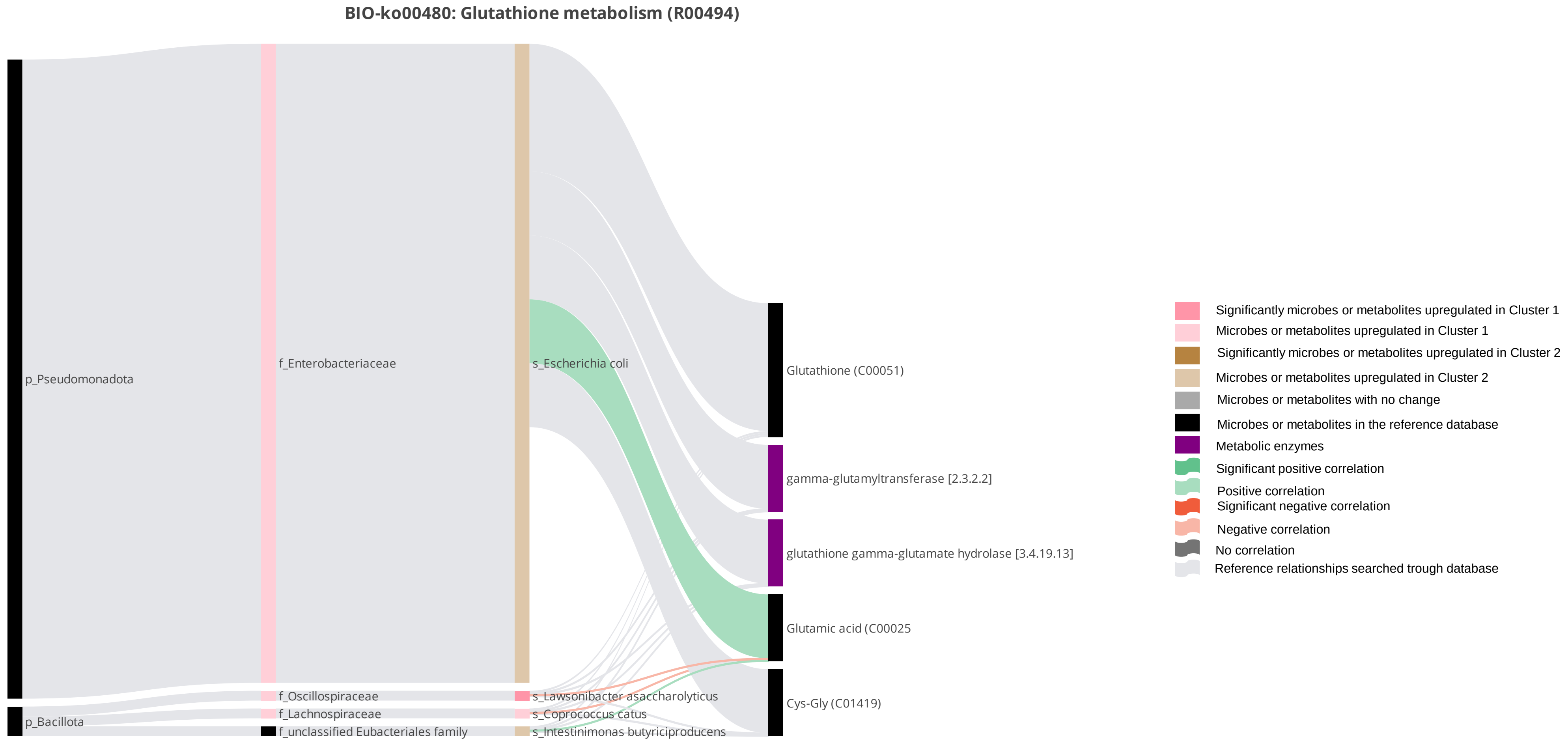

Supplementary figure 4S: Sankey Network diagram showing the identified microbes in the samples of the gut microbiome of patients from the study and their connection with the significant metabolic pathways from microbial and hos co-metabolism after MPEA analysis and the reactions involved.

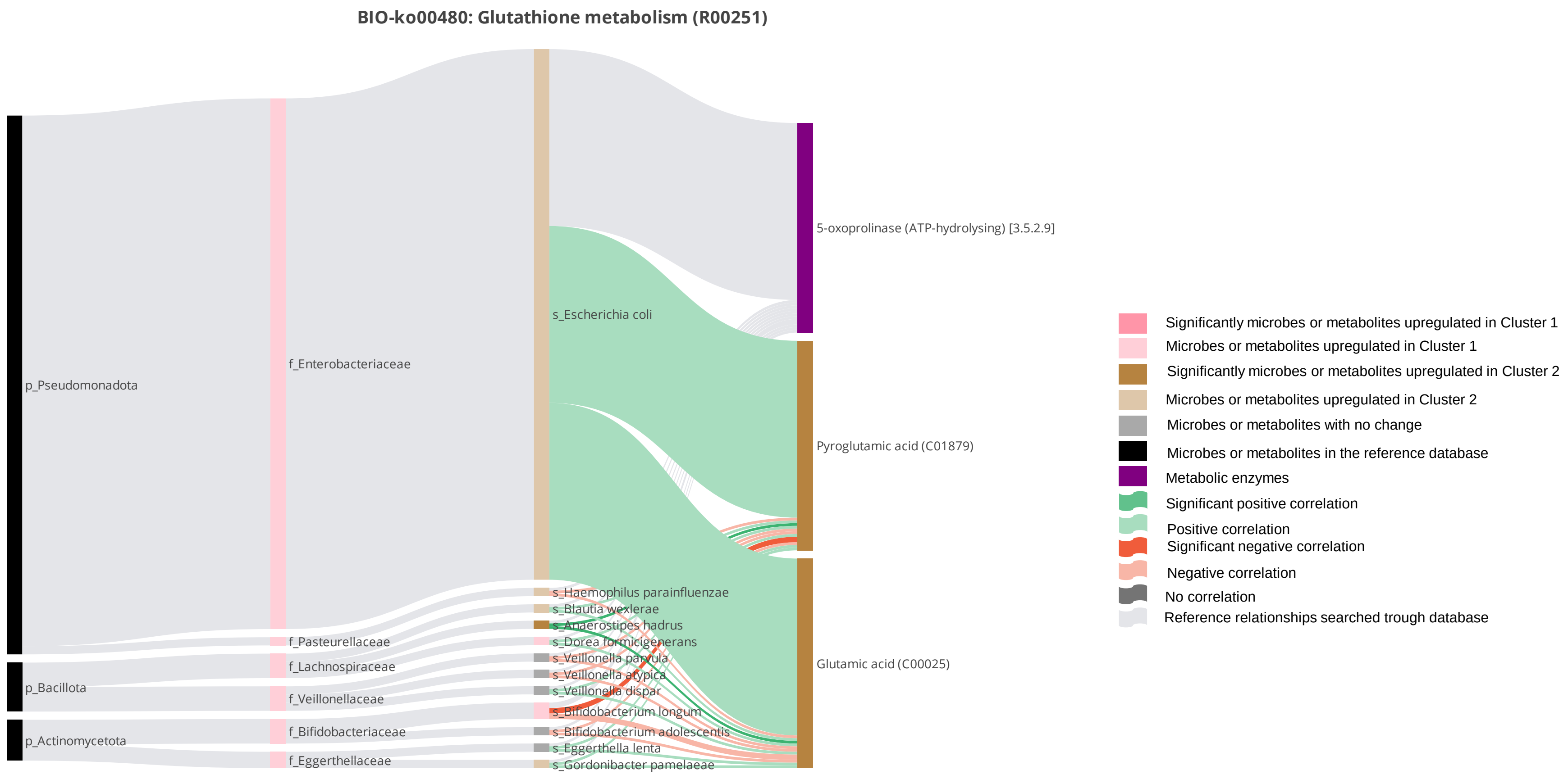

Supplement: Figure S4 — Sankey network diagram. [file msystems.00143-25-s0006.pdf]
