# Supplementary material for: Microbiome gut community structure and functionality are associated with symptom severity in non-responsive celiac disease patients undergoing a gluten-free diet
Source: mSystems. 2025 Jun 6;10(7):e00143-25. doi: 10.1128/msystems.00143-25 (PMC12282095; doi:10.1128/msystems.00143-25)
Supplement: Supplemental legends — Legends for supplemental material. [file msystems.00143-25-s0007.docx]

**Supplemental Data 1.** Strengthening The Organization and Reporting of Microbiome Studies (STORMS) checklist for reporting data used in the current study.

**Supplemental Data S2.** Metabolites identified by UHPLC-Q/TOF-MS/MS in positive and negative modes. Including m/z (mass-to-charge ratio), retention time in minutes (Rt), metabolite name, adduct type and identification type.

**Supplementary Data S3. A)** Alpha diversity values computed for each sample. **B)** Linear discriminant analysis effect size (LEfse) results showing taxon’s taxon table and LDA score from tCD, low-NRCD, and high-NRCD patients. **C)** Differential abundance analysis calculation between low-NRCD and high-NRCD patients made with the Mann-Whitney Test.

**Supplementary Data S4. A)** Table showing the total number of metabolites from different origins, including Human Metabolome Database (HMDB) and Kyoto Encyclopedia of Genes and Genomes (KEGG) identification numbers. **B)** Differential analysis of metabolites from low-NRCD and high-NRCD patients made with the Mann-Whitney Test. **C)** Metabolic pathway enrichment analysis (MPEA) according to the differential metabolites from co-metabolism. **D)** Metabolic pathway enrichment analysis (MPEA) according to the differential metabolites from microbiota.

**Supplementary Figure 1:** PRISMA flowchart summarizing the process to select metagenomics publicly available data from CD asymptomatic patients .

**Supplementary Figure 2A:** Plots of variables in multiple factor analysis (A) Group representation and (B) Circle plot showing the quantitative variables contribution.

**Supplementary Figure 2B.** Scree plot to select the variables to retain (A), and contribution of variables to dimension one (B), two (C) and three (D).

**Supplementary Figure 3** (A) Multidimensional scaling (MDS) of the Unifrac distance representing beta diversity before batch effect correction of the samples (B) Same as A but after batch effect correction.

**Supplementary figure 4:** Sankey Network diagram showing the identified microbes in the samples of the gut microbiome of patients from the study and their connection with the significant microbial metabolic pathways after MPEA analysis and the reactions involved.
